# Incidence and Outcomes of Vertebral Compression Fracture Among Patients Infected with COVID-19

**DOI:** 10.3390/jcm13247830

**Published:** 2024-12-22

**Authors:** Helen Zhang, Mariah Balmaceno-Criss, Abigail M. Fruge, Patrick A. Massey, Alan H. Daniels, Andrew S. Zhang

**Affiliations:** 1 Department of Orthopaedic Surgery, The Warren Alpert Medical School, Brown University, Providence, RI 02903, USA; helen_zhang2@brown.edu (H.Z.); mariah_balmaceno-criss@brown.edu (M.B.-C.); alan_daniels@brown.edu (A.H.D.); 2 School of Medicine, Louisiana State University Health Sciences Center, Shreveport, LA 71130, USA; amf002@lsuhs.edu; 3 Department of Orthopaedic Surgery, Louisiana State University Health Sciences Center, Shreveport, LA 71130, USA; patrick.massey@lsuhs.edu

**Keywords:** COVID-19, bone mineral density, pathological fractures, vertebral compression fracture, compression fracture incidence

## Abstract

**Background/Objectives**: Early studies have suggested that the SARS-CoV-2 virus has a deleterious effect on bone mineral density and may increase the risk of pathological fractures. This study characterized vertebral compression fractures in patients with and without a prior diagnosis of COVID-19. **Methods**: Using a nationwide claims database, this retrospective study used ICD-10 billing codes to identify patients with a diagnosis of vertebral compression fracture from January 2020 to April 2022. Two cohorts were created based on whether the patients had a concurrent diagnosis of COVID-19. Patient demographics, comorbidities, and outcome measures were characterized by descriptive analysis. **Results**: In total, 413,425 patients met the inclusion criteria. Among them, a total of 23,148 patients (5.60%) had a diagnosis of COVID-19 at the time of their compression fracture. Among the COVID-19 patients, the incidences of vertebral compression fracture were 0.42% in 2020 and 0.33% in 2021, in comparison to the historical average yearly incidence of 0.17% across all patients. The patients with COVID-19 at the time of compression fracture diagnosis had a higher rate of vitamin D deficiency (OR: 1.25) and a lower rate of routine healing (OR: 0.61). The patients without COVID-19 were more likely to be osteoporotic (OR: 0.88), experience additional compression fractures (OR: 0.38), and have kyphoplasty or vertebroplasty (OR: 0.73). **Conclusions**: Despite lower rates of osteoporosis, patients with a concomitant COVID-19 diagnosis exhibited a higher incidence of compression fractures. Although more research is needed, these results support increasing bone health surveillance in patients with a history of COVID-19 infection.

## 1. Introduction

The SARS-CoV-2 virus, which is responsible for COVID-19 infections, created a global pandemic that led to an unprecedented number of challenges within healthcare systems. In the aftermath of the pandemic, research has sought to understand both the acute and long-term impacts of COVID-19 on various organs. With regards to the skeletal system, previous studies have associated COVID-19 infection with direct dysregulation of bone homeostasis through inhibition of osteoblasts and activation of osteoclasts [1,2,3,4]. A pro-inflammatory response against viral infection has been correlated with indirect disruption of bone micro-architecture [5,6,7,8,9,10,11]. Alterations in bone mineralization and metabolism among patients previously infected with SARS-CoV-2 may place them at a heightened risk of secondary osteoporosis, joint destruction, and pathological stress fractures.

The contribution of viral infection to bone pathology is a relatively unexplored topic that has recently piqued scientific investigation, notably regarding viral influence on inflammatory disease, arthropathies, and infections of bones and joints. Viruses of interest include but are not limited to parvovirus B19, hepatitis B and C, human immunodeficiency virus (HIV), and alphaviruses [12]. For example, HIV-1 has previously been found to infect osteoclasts directly, thereby modifying bone resorption mechanisms [13]. The SARS-CoV-2 virus has been linked to elevated inflammatory markers such as C-reactive protein, IL-6, and TNF-α. Such elevations have been correlated with disease severity, with the aforementioned inflammatory markers mediating osteoblast regulation, receptor activator of nuclear factor-κB ligand (RANKL) signaling pathways, and cytokine storm induction [4,14,15]. Micro-architectural alterations in bone have also been reported as effects of SARS-CoV-2 infection. Such remodeling is evidenced at the lacunar level, potentially inducing osteopenic or osteoporotic conditions with decreased bone mineral density (BMD).

Patients with COVID-19 may have additional risk factors for bone fragility. Vitamin D deficiency and prolonged glucocorticoid therapy are factors noted to play a role in COVID-19 infection that may contribute to poor bone health. Immobilization is another factor, driving rapid losses of muscle mass and strength [16]. Frailty and chronic inflammation are likely to further deteriorate a patient’s health status and limit recovery. These findings indicate the importance of long-term monitoring during recovery from COVID-19 infection, including potential vitamin D supplementation and anti-osteoporosis therapy for those who require long-term corticosteroid treatment [14].

Compression fractures are a hallmark of osteoporosis commonly seen in elderly populations. Given the deleterious effects of the SARS-CoV-2 virus on BMD, a rise in osteoporotic compression fractures associated with the COVID-19 pandemic might be expected. As the treatment of osteoporosis and osteoporosis-related pathologies is projected to cost USD 25.3 billion annually in the United States by 2025, such a rise in the fracture rate could significantly impact the healthcare system nationally [17]. Vertebral compression fractures are especially notable, as they are the most common type of osteoporotic fracture and can significantly impact quality of life. Such fractures not only increase mortality but also the risk of subsequent compression fractures [18]. The European Prospective Osteoporosis Study (EPOS) previously estimated the annual incidences of vertebral compression fractures to be 10.7 per 1000 women and 5.7 per 1000 men [19]. A cross-sectional study using data from the 2019 Global Burden of Disease (GBD) Study found 8.6 million incident cases of vertebral fractures, 5.3 million prevalent cases, and 0.55 million age-standardized years lived with disability (YLDs) due to vertebral fracture [20]. Compared with 1990, the numbers of incident cases and YLDs had increased by 38% and 75%, respectively, in 2019.

Recent studies have examined osteoporotic fracture rates among the elderly following the onset of the COVID-19 pandemic [21]. However, these have been single-center studies that have strictly evaluated the epidemiology of fractures during the pandemic but not the resultant impact of the virus itself on subsequent fracture healing. Hence, the purpose of this study was to examine osteoporotic vertebral compression fracture rates and healing patterns among patients with and without a prior diagnosis of COVID-19 in the United States in order to elucidate the long-term repercussions of the SARS-CoV-2 virus on bone health.

## 2. Materials and Methods

### 2.1. Study Setting

This was a retrospective cohort study of patients identified through the national all-payer PearlDiver Mariner database (PearlDiver, Inc., Colorado Springs, CO, USA). This dataset contains longitudinal clinical data for over 161 million patients in the United States from January 2010 through April 2022. International Classifications of Diseases (ninth (ICD-9) and tenth (ICD-10) revisions) and Current Procedural Terminology (CPT) billing codes tied to insurance claims were used to track medical and surgical diagnoses, procedures, healthcare costs, and prescription medication data. This study was exempt from Institutional Review Board (IRB) review since no protected health information (PHI) was collected.

### 2.2. Participant Selection

Patient records were queried between 2020 and 2022 to identify adult patients with an ICD-10 diagnosis of osteoporotic vertebral compression fracture. Patients with a history of bone cancer, metastatic cancer, spinal neoplasm, or spinal infection were excluded. In addition, patients with reported trauma in the 30 days prior to their compression fracture claims were excluded. For patients with multiple instances of compression fracture within the specified time period, only the first instance was selected. Two cohorts were then created, representing patients with and without an existing diagnosis of COVID-19 at the time of the initial compression fracture. The ICD-10 codes for diagnosis of COVID-19 included a prior history of COVID-19 and post-COVID-19 disorder. All billing codes used are listed in Table S1.

### 2.3. Data Collection

Demographic, comorbidity, and outcome data were extracted. The comorbidities evaluated included obesity, morbid obesity, alcohol abuse, tobacco use, depression, diabetes, osteoarthritis, osteoporosis, use of osteoporosis drugs (including bisphosphonates, estrogen modulators, anabolic therapy, denosumab, and calcitonin), vitamin D deficiency, and steroid use (long-term). The outcome measures included healing (routine, delayed, non-union, and sequela) within 90 days after compression fracture, subsequent initial diagnosis of compression fracture, and subsequent kyphoplasty or vertebroplasty.

### 2.4. Statistical Analyses

Descriptive analysis was performed to characterize the patient demographics and comorbidities, and Student’s *t*-test and chi-square analyses were utilized to compare them across the experimental and control groups. For each comparison, an odds ratio (OR) and a 95% confidence interval (CI) were reported. A *p*-value of <0.05 was designated a priori to represent statistical significance. All statistical analyses were performed using the R Statistical Package (v4.2.1; R Core Team 2022, Vienna, Austria) within PearlDiver. To calculate the historical incidence of compression fractures, cases per year values were compared to the total number of patients within the database. For the years 2020–2021, the incidence of compression fracture was calculated separately for the COVID-19 and non-COVID-19 cohorts. For each patient in the 2020–2021 study sample, only the first occurrence of compression fracture was recorded for incidence calculation.

## 3. Results

In total, 413,425 patients met the inclusion criteria and had a diagnosis of vertebral compression fracture during 2020–2022. Of these patients, 23,148 (5.60%) had an existing diagnosis of COVID-19 at the time of their initial vertebral compression fracture.

### 3.1. Patient Characteristics

**Figure 1:**
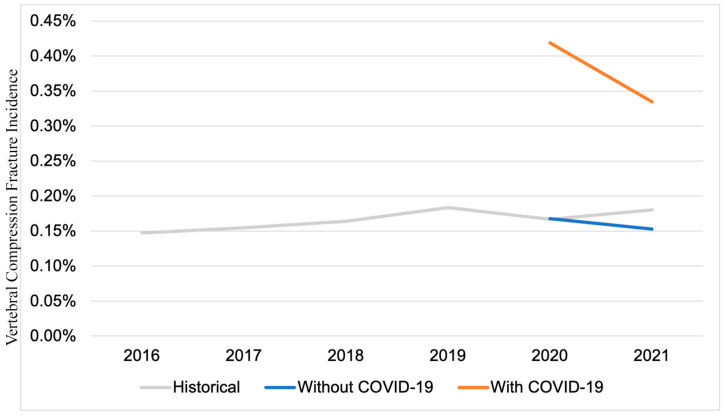
A comparison of historical compression fracture incidence. The incidence of initial compression fracture within the study period of 2020–2021 is shown for patients without and with a previous/existing COVID-19 diagnosis.

Across the two groups, age, sex, and regional distribution were relatively similar (Table 1). Between 2020 and 2022, the patients with COVID-19 had a vertebral compression fracture incidence of 0.26%, as opposed to the historical average annual incidence of 0.17% in all patients. In both 2020 and 2021, the incidence of compression fracture was higher in patients with a diagnosis of COVID-19 than in those without (2020: 0.42% vs. 0.17%, *p* < 0.001; 2021: 0.33% vs. 0.15%, *p* < 0.001) (Figure 1).

### 3.2. Comparison of Comorbidities

The patients with concomitant COVID-19 had higher Charlson Comorbidity Index (CCI) scores than those without COVID-19 (COVID-19 = 2.99 ± 2.43 versus non-COVID-19 = 2.62 ± 2.21, *p* < 0.001) (Table 2). Specifically, the COVID-19 patients had higher rates of obesity (OR = 1.50, 95% CI = 1.46–1.54, *p* < 0.001), morbid obesity (OR = 1.66, 95% CI = 1.59–1.74, *p* < 0.001), diabetes (OR = 1.46, 95% CI = 1.42–1.50, *p* < 0.001), osteoarthritis (OR = 1.13, 95% CI = 1.10–1.16, *p* < 0.001), vitamin D deficiency (OR = 1.25, 95% CI = 1.21–1.28, *p* < 0.001), and long-term (current) steroid use (OR = 1.62, 95% CI = 1.57–1.69, *p* < 0.001). The non-COVID-19 patients had higher rates of diagnosed osteoporosis (OR = 0.88, 95% CI = 0.86–0.90, *p* < 0.001) and use of osteoporosis drugs (OR = 0.80, 95% CI = 0.78–0.83, *p* < 0.001).

### 3.3. Fracture Healing

In terms of patient outcomes following compression fracture, the COVID-19 patients had a lower rate of routine healing (OR = 0.61, 95% CI = 0.57–0.64, *p* < 0.001), whereas the non-COVID-19 patients had higher rates of delayed healing (OR = 0.54, 95% CI = 0.45–0.65, *p* < 0.001) and sequela healing (OR = 0.57, 95% CI = 0.50–0.65, *p* < 0.001) (Table 3). The rate of non-union healing was not statistically different between the groups (OR = 1.04, 95% CI = 0.73–1.49, *p* = 0.823). The non-COVID-19 patients also had higher rates of additional compression fracture (OR = 0.38, 95% CI = 0.36–0.41, *p* < 0.001) and kyphoplasty/vertebroplasty (OR = 0.73, 95% CI = 0.69–0.76, *p* < 0.001).

## 4. Discussion

Given the known impact of the SARS-CoV-2 virus on bone homeostasis, this study aimed to characterize the rate of vertebral compression fractures and fracture healing patterns in patients with COVID-19 infections. Higher rates of vertebral compression fractures were found in patients with a prior diagnosis of COVID-19, specifically during the height of the pandemic, and these rates exceeded all historical yearly incidences. Higher rates of vitamin D deficiency, but lower rates of osteoporosis and utilization of osteoporosis medications, were also noted in this patient population. Finally, lower rates of routine healing were observed in patients with COVID-19 experiencing vertebral compression fractures in comparison to patients without a concurrent infection.

The deleterious effect of COVID-19 infection on bone mineral density has been reported in previous studies [5,6,22,23,24]. Despite this relationship, there is a paucity of research on its clinical implications, such as increased risk of fragility fractures. Di Filippo et al. conducted a retrospective cohort study of COVID-19 patients and reported thoracic vertebral fractures in 36% of patients [15]. However, since their study lacked a control group, it is unclear whether their findings reflect a higher rate of vertebral compression fractures in the cohort or increased detection. Battisti et al. assessed vertebral fractures by computed tomography (CT) in a cohort of 501 patients admitted to an emergency department during the early pandemic, finding similar prevalences in patients with and without COVID-19 [25]. They found that vertebral fractures were only associated with increased mortality risk in those without COVID-19. A single-center study from Lotan et al. did not find a significant difference in the overall incidence rate of vertebral compression fractures in 2020 compared to the two previous years [21]. The current study has a similar finding, noting similar overall incidence rates in the years before and after the COVID-19 pandemic. However, when the patients were stratified by the presence of COVID-19 infection, there were substantial differences between the two groups, which suggests that the SARS-CoV-2 virus may in fact have altered bone homeostasis and osteoporosis-related pathologies.

In particular, the incidence of vertebral compression fractures was higher in both 2020 and 2021 among the patients with COVID-19 compared to the patients without infections, with these rates surpassing all historical incidence rates. The COVID-19 patients, on average, developed compression fractures about 3 months (101 days) after their COVID-19 diagnosis. Since this is long after the acute phase of the infection, this may represent a long-term SARS-CoV-2-mediated disruption of bone metabolism. This hypothesis is supported by Elmedany et al., who found that patients with COVID-19 exhibited a significant decrease in bone mineral density in the lumbar spine 9 months post-infection compared to baseline [24]. The time to fracture onset also indicates that this finding is not merely an incidental imaging finding resulting from higher surveillance in COVID-19 patients presenting to the hospital. The time difference between diagnoses may also be due in part to delays in healthcare access.

Interestingly, the patients with COVID-19 suffering from vertebral compression fractures had lower rates of osteoporosis and osteoporosis medication use compared to the patients without COVID-19. However, the patients with concurrent COVID-19 at the time of fracture had higher rates of vitamin D deficiency and steroid usage, which both compromise bone health and place patients at higher risk for subsequent fragility fractures in the face of long-term COVID-19 sequelae. This could suggest that all else being equal, patients who contract COVID-19 may develop bone loss at a heightened rate, or subsequent treatment may predispose them to bone loss. In the short term, Berktas et al. found that hospitalized COVID-19 patients have multiple predilections for bone loss such as ages over 50, decreased mobility, malnutrition, hypocalcemia, and increased serum pro-inflammatory cytokines [23]. Thus, when patients with pre-existing factors that negatively impact bone health contract COVID-19, compression fractures that occur acutely may represent an accumulation of negative hits to the bone surpassing a critical threshold. However, it is possible that lower rates of osteoporosis and osteoporosis medication use are indicative of underdiagnosis or misclassification in this cohort.

The patients in our study’s COVID-19 population demonstrated lower rates of routine healing, which is supported by COVID-19 infection resulting in altered bone homeostasis. While we expected this population to also have higher rates of delayed and non-union healing, this was not reflected in this study’s findings, although they could have been misrepresented by coding disparities. Additionally, this could be a result of the hardships encountered when seeking in-office follow-up during the height of the pandemic. Finally, our COVID-19 cohort had lower rates of surgical management through kyphoplasty/vertebroplasty, illustrating the more tenuous clinical states of COVID-19-positive patients compared to non-COVID-19 patients, who are able to address their compression fractures in an elective setting and are eligible for cementation procedures without other precluding health factors. A poor clinical condition due to COVID-19-related illness may result in the deferment of surgical management to the point where it is no longer clinically indicated. Thus, limitation of surgical intervention due to the severity of COVID-19 is likely multifactorial.

The limitations of this study include the retrospective nature of the PearlDiver database. Retrospective studies are inherently limited in terms of potential bias, as well as a lack of causality and generalizability. The validity of our findings is reliant upon accurate documentation within the PearlDiver dataset. As claims data are used for billing and reimbursements, they may not contain all relevant conditions and outcomes. Mortality and long-term outcomes were not evaluated due to the limitations of the dataset and the recency of the COVID-19 pandemic. Variability in disease severity was not reflected by the data, which was instead considered a categorical variable. However, the comorbidity burden was thoroughly evaluated to account for the lack of these measurements. The higher rates of vertebral compression fractures and comorbidities in the COVID-19 patients may have been due to incidental diagnosis during screening. Conversely, the rates may have been lower than expected due to the overwhelming burden placed on the healthcare system at the height of the COVID-19 pandemic, during which precedence was placed on respiratory conditions rather than musculoskeletal conditions. Additionally, only the first instance of vertebral compression fracture was included for each patient in order to limit confounding and ambiguity regarding COVID-19 status in patients with multiple fractures. However, this may have limited our understanding of variable presentations and outcomes accompanying recurrent fractures.

## 5. Conclusions

Exposure to the SARS-CoV-2 virus and subsequent diagnosis of COVID-19 have been shown to have deleterious effects on BMD. The results of this study show that patients with COVID-19 experienced greater incidence of compression fractures, consistent with scenarios where BMD is compromised. Although more long-term follow-up is needed, practitioners should consider increasing surveillance of bone health in post-COVID-19 patients, who may be more susceptible to compression fractures due to viral exposure. Additional strategies for fracture prevention may include addressing underlying conditions, vitamin D supplementation, anti-osteoporosis therapies, and corticosteroid use stewardship.

## Figures and Tables

**Table 1:** Demographics of COVID-19 versus non-COVID-19 compression fracture patients. Age and CCI are reported with standard deviations. Otherwise, all data are reported as numbers and percentages of totals.

		COVID-19 N = 23,148	Non-COVID-19 N = 390,277	*p*-Value
Age, years		70.06 ± 13.39	70.69 ± 13.42	<0.001
CCI		2.99 ± 2.43	2.62 ± 2.21	<0.001
Female Sex		15,068 (65.1)	259,076 (66.4)	<0.001
Region				<0.001
	Midwest	6213 (26.8)	98,012 (25.1)	
	Northeast	4310 (18.6)	65,844 (16.9)	
	South	9387 (40.6)	155,383 (39.8)	
	West	3360 (14.5)	68,095 (17.4)	
	Unknown	191 (0.8)	2943 (0.7)	
	Midwest	6213 (26.8)	98,012 (25.1)	
Year				
2020	Total	6590 (28.4)	179,425 (45.9)	
	2020Q1	34 (0.1)	55,496 (14.2)	
	2020Q2	1209 (5.2)	37,337 (9.6)	
	2020Q3	1686 (7.3)	45,896 (11.8)	
	2020Q4	3661 (15.8)	40,696 (10.4)	
2021	Total	12,308 (53.2)	162,942 (41.7)	
	2021Q1	4019 (17.4)	40,258 (10.3)	
	2021Q2	2536 (11.0)	43,664 (11.2)	
	2021Q3	2840 (12.3)	40,730 (10.4)	
	2021Q4	2913 (12.6)	38,263 (9.8)	
2022	–	–	–	
	2022Q1	3330 (14.4)	35,762 (9.2)	
	2022Q2	920 (4.0)	12,175 (3.1)	
Time Between		101.17 ± 143.24	–	

Abbreviations: CCI = Charlson Comorbidity Index.

**Table 2:** Comparison of comorbidities among COVID-19 and non-COVID-19 compression fracture patients.

	COVID-19 (N = 23,148)	Non-COVID-19 (N = 390,277)	OR	95% CI	*p*-Value
Normal BMI	6439 (27.8)	100,588 (25.8)	1.11	1.08–1.14	<0.001
Obesity	9094 (39.3)	117,445 (30.1)	1.50	1.46–1.54	<0.001
Morbid Obesity	2457 (10.6)	26,012 (6.7)	1.66	1.59–1.74	<0.001
Alcohol Abuse	2714 (11.7)	43,435 (11.1)	1.06	1.02–1.11	0.005
Depression	12,281 (53.1)	179,375 (46.0)	1.33	1.29–1.36	<0.001
Diabetes	11,315 (48.9)	154,615 (39.6)	1.46	1.42–1.50	<0.001
Osteoarthritis	11,040 (47.7)	174,702 (44.8)	1.13	1.10–1.16	<0.001
Osteoporosis	10,009 (43.2)	181,042 (46.4)	0.88	0.86–0.90	<0.001
Osteoporosis Drugs	5783 (25.0)	114,548 (29.4)	0.80	0.78–0.83	<0.001
Vitamin D Deficiency	9173 (39.6)	134,663 (34.5)	1.25	1.21–1.28	<0.001
Steroid Use	3690 (15.9)	40,788 (10.5)	1.62	1.57–1.69	<0.001
Tobacco Use	11,356 (49.1)	179,673 (46.0)	1.13	1.10–1.16	<0.001

Abbreviations: BMI = Body Mass Index, OR = odds ratio, CI = confidence interval.

**Table 3:** Comparison of fracture healing outcomes among COVID-19 and non-COVID-19 compression fracture patients.

	COVID-19 (N = 23,148)	Non-COVID-19 (N = 390,277)	OR	95% CI	*p*-Value
Routine Healing	1087 (4.7)	29,359 (7.5)	0.61	0.57–0.64	<0.001
Delayed Healing	116 (0.5)	3629 (0.9)	0.54	0.45–0.65	<0.001
Non-union Healing	32 (0.1)	518 (0.1)	1.04	0.73–1.49	0.823
Sequela Healing	221 (1.0)	6494 (1.7)	0.57	0.50–0.65	<0.001
Additional Compression Fracture	1054 (4.6)	43,208 (11.1)	0.38	0.36–0.41	<0.001
Kyphoplasty/Vertebroplasty	1750 (7.6)	39,534 (10.1)	0.73	0.69–0.76	<0.001

Abbreviations: OR = odds ratio, CI = confidence interval.

## Data Availability

Restrictions apply to the availability of these data. These data were obtained from the PearlDiver Mariner database (PearlDiver, Inc., Colorado Springs, CO, USA).

## Supplementary Materials

The following supporting information can be downloaded at https://www.mdpi.com/article/10.3390/jcm13247830/s1, Table S1: CPT, ICD-9, and ICD-10 codes used to define each cohort.
