# Supplementary material for: Incidence and Outcomes of Vertebral Compression Fracture Among Patients Infected with COVID-19
Source: J Clin Med. 2024 Dec 22;13(24):7830. doi: 10.3390/jcm13247830 (PMC11680045; doi:10.3390/jcm13247830)
Supplement: Supplementary file 1 [file jcm-13-07830-s001.zip › jcm-3327563-supplementary.pdf]

Table S1: CPT, ICD-9, and ICD-10 codes used to define each cohort.

Description	Codes Used
Compression Fracture	ICD-10-D-S22000A, ICD-10-D-S22000B, ICD-10-D-S22010A, ICD-10-D-S22010B, ICD-10-D-S22020A, ICD-10-D-S22020B, ICD-10-D-S22030A, ICD-10-D-S22030B, ICD-10-D-S22040A, ICD-10-D-S22040B, ICD-10-D-S22050A, ICD-10-D-S22050B, ICD-10-D-S22060A, ICD-10-D-S22060B, ICD-10-D-S22070A, ICD-10-D-S22070B, ICD-10-D-S22080A, ICD-10-D-S22080B, ICD-10-D-S32000A, ICD-10-D-S32000B, ICD-10-D-S32010A, ICD-10-D-S32010B, ICD-10-D-S32020A, ICD-10-D-S32020B, ICD-10-D-S32030A, ICD-10-D-S32030B, ICD-10-D-S32040A, ICD-10-D-S32040B, ICD-10-D-S32050A, ICD-10-D-S32050B, ICD-10-D-M4850XA, ICD-10-D-M4851XA, ICD-10-D-M4852XA, ICD-10-D-M4853XA, ICD-10-D-M4854XA, ICD-10-D-M4855XA, ICD-10-D-M4856XA, ICD-10-D-M4857XA, ICD-10-D-M4858XA, ICD-10-D-M8008XA, ICD-10-D-M8088XA
COVID-19	ICD-10-D-U071, ICD-10-D-U072, ICD-10-D-U089, ICD-10-D-U099, ICD-10-D-U109, ICD-10-D-Z8616
Normal BMI	ICD-9-D-V851, ICD-10-D-Z6820, ICD-10-D-Z6821, ICD-10-D-Z6822, ICD-10-D-Z6823, ICD-10-D-Z6824, ICD-9-D-V8521, ICD-9-D-V8522, ICD-9-D-V8523, ICD-9-D-V8524, ICD-9-D-V8525, ICD-10-D-Z6825, ICD-10-D-Z6826, ICD-10-D-Z6827, ICD-10-D-Z6828, ICD-10-D-Z6829

Obesity	ICD-10-D-Z6830, ICD-10-D-Z6831, ICD-10-D-Z6832, ICD-10-D-Z6833, ICD-10-D-Z6834, ICD-10-D-Z6835, ICD-10-D-Z6836, ICD-10-D-Z6837, ICD-10-D-Z6838, ICD-10-D-Z6839, ICD-9-D-V8530, ICD-9-D-V8531, ICD-9-D-V8532, ICD-9-D-V8533, ICD-9-D-V8534, ICD-9-D-V8535, ICD-9-D-V8536, ICD-9-D-V8537, ICD-9-D-V8538, ICD-9-D-V8539
Morbid Obesity	ICD-10-D-Z6841, ICD-10-D-Z6842, ICD-10-D-Z6843, ICD-10-D-Z6844, ICD-10-D-Z6845, ICD-9-D-V8541, ICD-9-D-V8542, ICD-9-D-V8543, ICD-9-D-V8544, ICD-9-D-V8545
Depression	ICD-9-D-29620, ICD-9-D-29621, ICD-9-D-29622, ICD-9-D-29623, ICD-9-D-29624, ICD-9-D-29630, ICD-9-D-29631, ICD-9-D-29632, ICD-9-D-29633, ICD-9-D-29634, ICD-9-D-29682, ICD-9-D-311, ICD-9-D-3004, ICD-10-D-F33
Osteoporosis	ICD-9-D-73300, ICD-9-D-73301, ICD-9-D-73302, ICD-9-D-73303, ICD-9-D-73309, ICD-10-D-M810, ICD-10-D-M818, ICD-10-D-M8008XA, ICD-10-D-M8088XA, ICD-10-D-M8000XA, ICD-10-D-M8008XD, ICD-10-D-Z87310
Vitamin D Deficiency	ICD-9-D-2689, ICD-10-D-E559
Steroid Use	ICD-10-D-Z7951, ICD-10-D-Z7952
Routine Healing	ICD-10-D-S22000D, ICD-10-D-S22010D, ICD-10-D-S22020D, ICD-10-D-S22030D, ICD-10-D-S22040D, ICD-10-D-S22050D, ICD-10-D-S22060D, ICD-10-D-S22070D, ICD-10-D-S22080D, ICD-10-D-S32000D, ICD-10-D-

	S32010D, ICD-10-D-S32020D, ICD-10-D-S32030D, ICD-10-D-S32040D, ICD-10-D-S32050D,ICD-10-D-M4850XD, ICD-10-D-M4851XD, ICD-10-D-M4852XD, ICD-10-D-M4853XD, ICD-10-D-M4854XD, ICD-10-D-M4855XD, ICD-10-D-M4856XD, ICD-10-D-M4857XD, ICD-10-D-M4858XD, ICD-10-D-M8008XD, ICD-10-D-M8088XD
Delayed Healing	ICD-10-D-S22000G, ICD-10-D-S22010G, ICD-10-D-S22020G, ICD-10-D-S22030G, ICD-10-D-S22040G, ICD-10-D-S22050G, ICD-10-D-S22060G, ICD-10-D-S22070G, ICD-10-D-S22080G, ICD-10-D-S32000G, ICD-10-D-S32010G, ICD-10-D-S32020G, ICD-10-D-S32030G, ICD-10-D-S32040G, ICD-10-D-S32050G,ICD-10-D-M4850XG, ICD-10-D-M4851XG, ICD-10-D-M4852XG, ICD-10-D-M4853XG, ICD-10-D-M4854XG, ICD-10-D-M4855XG, ICD-10-D-M4856XG, ICD-10-D-M4857XG, ICD-10-D-M4858XG, ICD-10-D-M8008XG, ICD-10-D-M8088XG
Nonunion Healing	ICD-10-D-S22000K, ICD-10-D-S22010K, ICD-10-D-S22020K, ICD-10-D-S22030K, ICD-10-D-S22040K, ICD-10-D-S22050K, ICD-10-D-S22060K, ICD-10-D-S22070K, ICD-10-D-S22080K, ICD-10-D-S32000K, ICD-10-D-S32010K, ICD-10-D-S32020K, ICD-10-D-S32030K, ICD-10-D-S32040K, ICD-10-D-S32050K,ICD-10-D-M4850XK, ICD-10-D-M4851XK, ICD-10-D-M4852XK, ICD-10-D-M4853XK, ICD-10-D-M4854XK, ICD-10-D-M4855XK, ICD-10-D-M4856XK, ICD-10-D-M4857XK, ICD-10-D-M4858XK, ICD-10-D-M8008XK, ICD-10-D-M8088XK
Sequela Healing	ICD-10-D-S22000S, ICD-10-D-S22010S, ICD-10-D-S22020S, ICD-10-D-S22030S, ICD-10-D-S22040S, ICD-10-D-S22050S, ICD-10-D-S22060S,

	ICD-10-D-S22070S, ICD-10-D-S22080S, ICD-10-D-S32000S, ICD-10-D-S32010S, ICD-10-D-S32020S, ICD-10-D-S32030S, ICD-10-D-S32040S, ICD-10-D-S32050S,ICD-10-D-M4850XS, ICD-10-D-M4851XS, ICD-10-D-M4852XS, ICD-10-D-M4853XS, ICD-10-D-M4854XS, ICD-10-D-M4855XS, ICD-10-D-M4856XS, ICD-10-D-M4857XS, ICD-10-D-M4858XS, ICD-10-D-M8008XS, ICD-10-D-M8088XS
Kyphoplasty, Vertebroplasty	CPT-22510:CPT-22515, CPT-22520:CPT-22525
